# Supplementary figures and images for: Transcriptome Analysis and Identification of Genes Associated with Cotton Seed Size
Source: Int J Mol Sci. 2024 Sep 11;25(18):9812. doi: 10.3390/ijms25189812 (PMC11432076; doi:10.3390/ijms25189812)

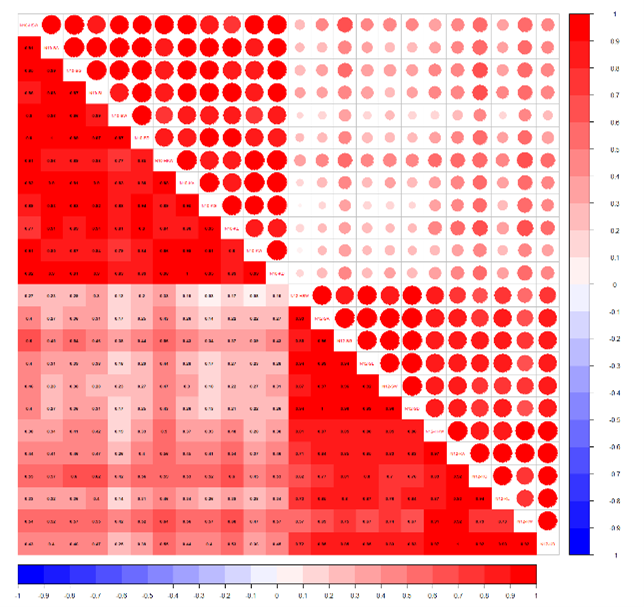

Supplement: Supplementary file 1 [file ijms-25-09812-s001.zip › Figure S1.tif]

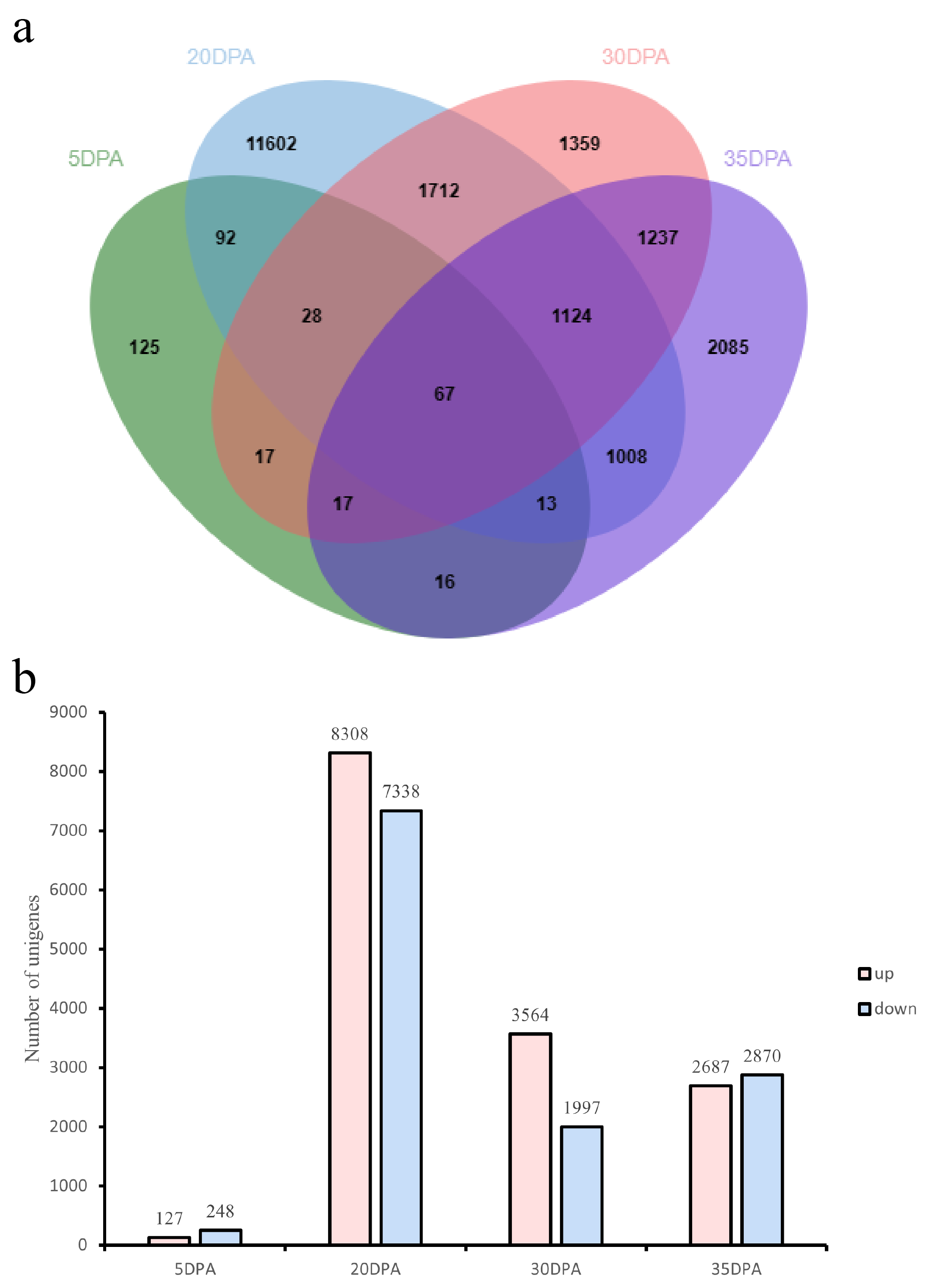

Supplement: Supplementary file 1 [file ijms-25-09812-s001.zip › Figure S2.tif]
